# Kinetic parameter prediction using neural networks identifies limitations to C_4_ photosynthesis

**DOI:** 10.1111/nph.71280

**Published:** 2026-05-28

**Authors:** Philipp Wendering, John Ferguson, Rudan Xu, Johannes Kromdijk, Zoran Nikoloski

**Affiliations:** ^1^ Department of Plant Science University of Cambridge Downing Street Cambridge CB2 3EA UK; ^2^ School of Life Sciences University of Essex Colchester CO4 3SQ UK; ^3^ Bioinformatics, Institute of Biochemistry and Biology University of Potsdam Karl‐Liebknecht‐Str. 24‐25 Potsdam 14476 Germany; ^4^ Systems Biology and Mathematical Modeling Max Planck Institute of Molecular Plant Physiology Am Mühlenberg 1 Potsdam 14476 Germany

**Keywords:** C_4_ photosynthesis, deep learning, gas exchange, kinetic model, parameterization

## Abstract

- Kinetic models of photosynthesis enable time‐resolved predictions of traits related to this key process and provide the means to identify factors limiting photosynthesis. However, the use of large‐scale models is currently limited by the lack of efficient approaches to estimate the hundreds of genotype‐specific kinetic parameters. Here, we present C4TUNE, an artificial neural network that can efficiently predict parameters of a large‐scale photosynthesis model from photosynthesis response curves.
- C4TUNE was trained on a biologically relevant synthetic dataset comprising matched samples of parameters and response curves obtained using a C_4_ photosynthesis kinetic model. To speed up the training of C4TUNE, we devised a surrogate neural network to predict photosynthesis response curves directly from the model parameters and environmental inputs.
- Given response curves as input, we showed that over 99% of the parameter vectors predicted by C4TUNE could be used directly in simulation of the kinetic model and resulted in excellent fits. Finally, we applied C4TUNE to predict parameters for a population of 68 maize genotypes across two seasons.
- The predicted genotype‐specific parameters allowed pinpointing factors that limit photosynthetic efficiency, validated using simulations. Therefore, the use of C4TUNE presents a fast and precise approach for parameter prediction based on minimal datasets.

## Graphical abstract

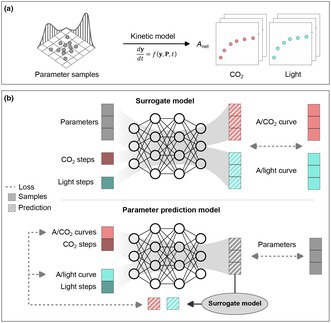

## Zusammenfassung

- Kinetische Photosynthesemodelle ermöglichen zeitlich aufgelöste Vorhersagen von Merkmalen, die mit diesem Schlüsselprozess zusammenhängen. Weiterhin bieten sie die Möglichkeit, Faktoren zu identifizieren, die Photosynthese limitieren. Der Einsatz großskaliger Modelle wird derzeit jedoch durch den Mangel an effizienten Ansätzen zur Schätzung der Hunderten von genotypspezifischen kinetischen Parametern eingeschränkt. Hier stellen wir C4TUNE vor, ein künstliches neuronales Netzwerk, das Parameter eines Photosynthesemodells anhand von Photosynthese‐Reaktionskurven effizient vorhersagen kann.
- C4TUNE wurde auf einem biologisch relevanten synthetischen Datensatz trainiert, der zusammengehöhrende Parameter und Reaktionskurven enthält, die mithilfe eines kinetischen C_4_‐Photosynthesemodells simuliert wurden. Um das Training von C4TUNE zu beschleunigen, haben wir ein Surrogat‐Modell entwickelt, welches die Photosynthese‐Reaktionskurven direkt aus den Modellparametern und experimentellen Bedingungen vorhersagt.
- Anhand der als Eingabe verwendeten Reaktionskurven konnten wir zeigen, dass über 99% der von C4TUNE vorhergesagten Parametervektoren direkt für Simulationen mithilfe des kinetischen Modells verwendet werden konnten und zu hervorragenden Übereinstimmungen führten. Schließlich wandten wir C4TUNE an, um Parameter für eine Population von 68 Mais‐Genotypen für zwei Vegetationsperioden vorherzusagen.
- Die vorhergesagten genotypspezifischen Parameter ermöglichten es, Faktoren zu identifizieren, die die Photosyntheseeffizienz limitieren; diese wurden durch Simulationen bestätigt. Somit bietet C4TUNE einen schnellen und präzisen Ansatz für die Parametervorhersage auf der Grundlage minimaler Datensätze.

## Introduction

Photosynthesis is a critical determinant of plant production, yet it is still far from its theoretical efficiency (Zhu *et al*., 2010). Photosynthetic efficiency is determined by multiple factors across different scales, from light interception to CO_2_ conversion and partitioning of carbon assimilates (Croce *et al*., 2024). Much effort has focused on engineering the core carbon metabolism, on minimizing losses of fixed CO_2_ via the photorespiratory pathway (South *et al*., 2019) as well as on the individual or combined manipulation of enzyme expression in the Calvin‐Benson cycle, electron transport, carbon transport, and photoprotection (Simkin *et al*., 2019). Despite the success of these experiment‐driven efforts, it remains challenging to identify what limits photosynthetic efficiency, particularly under rapidly changing environmental conditions projected for future climate scenarios (Dusenge *et al*., 2019; World Meteorological Organization (WMO), 2025). Therefore, a paradigm shift in the search for molecular factors that limit photosynthesis, and their manipulation via gene editing tools, is urgently needed to guide photosynthesis improvements and downstream biotechnological applications.

Kinetic models of photosynthesis offer a tractable way to identify molecular factors that limit photosynthesis across different environments. The existing kinetic models of C_3_ (Zhu *et al*., 2013; Kannan *et al*., 2019; Zhao *et al*., 2021) and C_4_ photosynthesis (Wang *et al*., 2014, 2021) comprise reactions involved in gas exchange, light absorption and carbon fixation, along with canonical pathways of sucrose and starch synthesis. These models detail the change in metabolite concentrations in terms of reaction fluxes using a system of ordinary differential equations (ODEs). The reaction fluxes are in turn described by the enzyme kinetics, comprising a combination of Michaelis–Menten, mass action, and convenience kinetics as well as empirical algebraic equations (Zhu *et al*., 2013; Kannan *et al*., 2019; Wang *et al*., 2021; Zhao *et al*., 2021). The enzyme kinetics relate metabolite and enzyme concentrations to the flux of a particular reaction using enzyme‐specific parameters (e.g. Michaelis–Menten constant, *K*_M_, and maximum reaction rate of an enzyme, *V*_max_). The kinetic parameters are properties of enzymes and can be manipulated by gene editing of the corresponding enzyme‐coding gene or other upstream regulatory components (Wang *et al*., 2025), providing a tangible means for testing and validating the identified factors limiting photosynthesis.

Data on kinetic parameters from a population of plants along with read‐outs of net photosynthesis rate offer the means to identify kinetic parameters strongly correlated to photosynthesis rate in a single or a set of environments. These parameters are candidates for factors that limit photosynthesis, as their manipulation in a specified direction, that is decrease for negatively correlated and increase for positively correlated, may be used to increase photosynthesis. However, obtaining data on kinetic parameters spanning all enzymes in photosynthesis‐related pathways is an experimentally unfeasible task, due to the time and resources required. Kinetic models of photosynthesis together with data on photosynthesis‐related traits can be used to arrive at model parameterizations following, in principle, two directions: (1) parameter estimation, by model fitting procedures, and (2) parameter prediction, by employing artificial intelligence (i.e. machine or deep learning) models to predict parameters, such that photosynthesis‐related traits simulated by the kinetic model match the measured traits. Genotype‐specific parameterization can in turn be used to identify parameters that limit photosynthesis following the previously described correlation‐based procedure.

The proposed direction is made tractable by advances in deep learning for model parameterization (Toumpe *et al*., 2025). Despite advances in approaches for parameter estimation (Schälte *et al*., 2023; Groves *et al*., 2024; Hu *et al*., 2024), such as those relying on Monte‐Carlo sampling, parameter estimation methods are time‐intensive, and their out‐of‐the‐box usage in biology is nontrivial due to the need for modeling skills. By contrast, recently developed deep learning approaches employ physics‐informed neural networks, where the ODEs are used to constrain the training process (Raissi *et al*., 2019; Cuomo *et al*., 2022), as well as generative models, that is, models trained to predict model parameterizations with defined characteristics without any input other than random noise (Choudhury *et al*., 2022, 2024). The proposed generative approaches facilitate the sampling of millions of model parameterizations with stable characteristics that agree with experimental observations in a matter of seconds. For model training, both approaches can also integrate various omics data by pre‐processing them into steady‐state profiles via thermodynamic stoichiometric modeling to obtain a set of reaction fluxes, metabolite concentrations, and thermodynamic parameters. To generate training data or evaluate model convergence properties using the steady‐state profiles, these approaches assume standard enzyme kinetics (Miskovic & Hatzimanikatis, 2010; Weilandt *et al*., 2023), not typical for models of photosynthesis (see previosly). Therefore, the parameter estimation in currently available large‐scale photosynthesis models poses additional challenges because they comprise combinations of enzyme kinetics and empirical equations, and due to the sparsity of data that can be used to validate model parameterizations.

Here we developed C4TUNE (C_4_ Tuning Engine), a deep learning approach that predicts complete parameterizations of a C_4_ photosynthesis model from gas exchange data that are routinely measured in photosynthesis research, that is, response curves of net CO_2_ assimilation rate, $A_{\mathrm{net}}$, to different ambient CO_2_ partial pressures and light intensities. C4TUNE uses synthetic, biologically constrained training data generated by kinetic model simulation of sampled parameter vectors. During model training, the biological relevance of the predicted parameters is ensured by: (1) respecting the covariance structure of the model parameters and (2) assessing the simulated $A_{\mathrm{net}}$ response curves. To enable time‐resolved response curve simulation using the predicted parameters during model training in reasonable time, we used a surrogate model trained to reproduce kinetic model simulations. To this end, we built a surrogate model for a large‐scale kinetic model of C_4_ photosynthesis (Wang *et al*., 2021) that comprises 236 tunable parameters for 123 reactions and 109 mass balances. As a result, the trained C4TUNE model efficiently predicts model parameters given $A_{\mathrm{net}}$ measurements as input. We demonstrated that by sampling inputs (with added noise) and subsequent parameter prediction, thousands of possible model parameterizations can be obtained in only few seconds. Finally, we applied C4TUNE to predict parameters for 68 genotypes of a multiple‐parent advanced generation intercross (MAGIC) maize population for two growing seasons. Using correlation‐based analysis, we pinpointed parameters that limit photosynthesis, raising candidates for finetuning of this key process. Therefore, C4TUNE provides the means for efficient and complete model parameterization, which can be used to derive valuable insights into limiting factors and enable simulations of genotype‐specific photosynthesis in unseen environments.

## Materials and Methods

### C_4_ kinetic model

We made use of a previously published C_4_ photosynthesis kinetic model (Wang *et al*., 2021) with minor modifications (Xu *et al*., 2025). The changes to the original model include updates to the equilibrium constants, $K_{\mathrm{eq}}$, which were inferred from thermodynamic data using the Equilibrator calculation tool (https://equilibrator.weizmann.ac.il, Beber *et al*., 2022). Moreover, all *K*_M_ and *K*_i_ values of the RuBisCO enzyme were unified between the carboxylation and oxygenation reactions, which were previously distinct between the two reactions in the model. This was done to ensure that only one set of kinetic constants was estimated for RuBisCO (Xu *et al*., 2025).

### Simulation of $A_{\mathrm{net}}$ response curves

All simulated response curves of $A_{\mathrm{net}}$ to changes in ambient CO_2_ or light intensity were performed while keeping constant all other environmental conditions accounted for in the model. More precisely, we set the ambient temperature to 25°C, wind speed was set to 3.5 m s^−1^, and relative humidity was set to 65%. The steps in ambient CO_2_ partial pressure for curve simulations were 400, 600, 800, 1000, 1250, 400, 300, 250, 200, 100, 75, 25 μbar at a constant light intensity of 1800 μmol photons m^−2^ s^−1^. For the simulations of light response curves, we used light intensities of 1800, 1100, 500, 300, 150, and 50 μmol photons m^−2^ s^−1^ at a constant ambient CO_2_ partial pressure of 400 μbar. The simulation time for the first and sixth timesteps was set to 3600 s to guarantee the establishment of a steady state in $A_{\mathrm{net}}$ at 400 μbar, while all other simulation intervals were 120 s long. Timepoint six was discarded after the simulation. Moreover, two stop criteria were used in the ODE solver: (1) when $A_{\mathrm{net}}$ reaches a steady state, and (2) when the simulation time on the machine exceeds 30 s. The second criterion was added to exclude models with low convergence times as well as to reduce the time required for individual simulations. These settings are equivalent to the ones used to estimate parameters for maize accessions in a related work (Xu *et al*., 2025). All simulations were carried out using Matlab's *ode15s* solver (MATLAB, 2024) with a relative tolerance of 10^−4^ and the requirement for all state variables to be non‐negative.

### Criteria for physiologically relevant model parameterizations

The following criteria were employed to classify kinetic models as relevant: (1) convergence of the ODE solver in the simulation of $A_{\mathrm{net}}$ response curves to both CO_2_ and light intensity, (2) in case the solver returned complex values, the imaginary parts must not exceed $10^{-9}$, (3) the simulated curve must contain at least one positive value, (4) all simulated values are between −10 μmol m^−2^ s^−1^ and 70 μmol m^−2^ s^−1^, and (5) no more than one change in monotonicity per response curve (only one change from increasing to decreasing direction or vice versa is allowed).

### Sampling the parameter space of the C_4_ photosynthesis model

The space of parameters associated with physiologically relevant kinetic models is multidimensional and shaped by non‐linear relationships between parameters (Choudhury *et al*., 2022; Borisyak *et al*., 2024). We assessed the model's sensitivity to perturbations of the initial parameter values (Wang *et al*., 2021) and found that many of the resulting kinetic models were infeasible or resulted in physiologically irrelevant $A_{\mathrm{net}}$ response curves. Therefore, we relied on previous work, where parameters were estimated for 68 maize (*Zea mays* L.) accessions over 2 yr (Supporting Information Notes S1; Xu *et al*., 2025). Based on these parameter estimates, $P_{\mathrm{acc}}$, 1000 random parameter samples, $P_{1}^{*}$, were drawn from a log‐normal distribution:

(Eqn 1) $$\log\left(P_{1}^{*}\right)=N\left(\mu_{\mathrm{acc}},\sigma_{\mathrm{acc}}\right),$$

where $\mu_{\mathrm{acc}}$ and $\sigma_{\mathrm{acc}}$ are the average and SD of the log‐transformed values in $P_{\mathrm{acc}}$ over all accessions. In total, 713 of the 1000 parameter samples resulted in physiologically relevant kinetic models. Using dimensionality reduction approaches, such as principal component analysis or t‐distributed neighbor embedding (t‐SNE) (van der Maaten & Hinton, 2008) it was not possible to distinguish between parameter vectors associated with relevant or irrelevant kinetic models. In a previous study, iterative application of rules derived from decision trees has been used to distinguish between relevant and irrelevant parameter vectors (Andreozzi *et al*., 2016). While the potential of employing such a rule‐based approach is an interesting avenue for future developments, we decided to use a more simplistic approach for this study, which assumes that the joint distribution of the relevant parameter can be captured by their covariance structure. To potentially increase the fraction of relevant parameter vectors, we used the covariance matrix ∑ of the log‐transformed relevant samples $P_{1,\mathrm{rel}.}^{*}$ to generate 10^6^ additional parameter samples that follow the same multivariate distribution:

(Eqn 2) $$\log\left(P_{2}^{*}\right)=\log\bar{P_{1,\mathrm{rel}.}^{*}}+\mathrm{LZ}$$

(Eqn 3) $$Z\sim N\left(0,1\right)$$

(Eqn 4) $$\sum =L^{T}L$$

The matrix L was obtained using Cholesky decomposition of ∑ (Eqn 4). By the multiplication of L and Z in Eqn 2, it is ensured that the resulting parameter samples $P_{2}^{*}$ have the mean and SD as well as preserved covariance structure as the samples in $P_{1,\mathrm{rel}.}^{*}$. Out of the additional 10^6^ samples, 764 091 were associated with physiologically relevant models, based on the criteria defined above. Compared to the first 1000 samples, the fraction of relevant parameter vectors increased from 71.3 to 76.4%. In total, we obtained 764 804 relevant samples for further analysis.

### Clustering of A/CO_2_ and A/light curves

To assess the diversity of simulated $A_{\mathrm{net}}$ response curves, a clustering analysis was performed on a random subset of 10 000 samples drawn from the generated dataset. For both curve types, the $A_{\mathrm{net}}$ values were first embedded using t‐SNE (van der Maaten & Hinton, 2008), followed by spectral clustering, using K‐medoids as the clustering algorithm based on Euclidean distance. The clustering quality was determined for different values of the number of clusters, 2≤K≤15, based on the median Silhouette Index (Fig. S1). For both curve types, the optimal K was chosen as the cluster number that resulted in a good visual separation, while yielding a Silhouette Index, which is close to the optimum for this curve type (K=12 for A/CO_2_ curves and K=7 for A/light curves, Figs S2, S3).

### Assessment of parameter identifiability

To quantify the identifiability of the parameter space, we determined the variability among parameter vectors that result in identical $A_{\mathrm{net}}$ response curves. To this end, we randomly selected 10 000 parameter sets as seeds and proceeded to identify curves in the complete dataset, which had a very low overall Canberra distance to the curves associated with the seed parameter vectors. The distance $d_{\mathrm{ij}}$ between two curves i and j was defined as

(Eqn 5) $$d_{\mathrm{ij}}=\frac{1}{n}\sum_{k=1}^{n}\frac{|A_{\mathrm{net}}\left(i,k\right)-A_{\mathrm{net}}\left(j,k\right)|}{\left|A_{\mathrm{net}}\left(i,k\right)\right|+\left|A_{\mathrm{net}}\left(j,k\right)\right|},$$

where n denotes the number of CO_2_ or light steps used to generate the curve. For each seed, we considered all response curves as identical if the average distance (Eqn 5) of the A/CO_2_ and A/light curves was below 0.01. For each cluster of identical curves, the coefficient of variation was calculated if the cluster size was greater or equal to five (*n* = 152).

We then determined the coefficient of variation (CV) across the parameter vectors associated with identical curves found for each of the seeds. Seeds for which we identified fewer than 10 identical curves were not considered. The CV is defined by:

(Eqn 6) $$\mathrm{CV}=\frac{\sigma }{\mu },$$

where μ and σ denote the mean and SD of the parameter values across a group of identical curve pairs.

### Neural network training

By inspection of the sampled kinetic parameters and associated $A_{\mathrm{net}}$ response curves, we found considerable variation among the parameters associated with identical curves (‘Assessment of parameter identifiability’ in the Materials and Methods section). In a machine learning setting, such many‐to‐one relations in the training data can be problematic and can hamper the learning process. Therefore, we reasoned that not only the distance between predicted parameter values, but also the curves resulting from the predicted parameters should enter the loss function guiding neural network training for parameter prediction. This arrangement ensures that the model does not only learn to predict the underlying parameters of the response curves, but also considers redundancy in the parameter space.

**Fig. 1:**
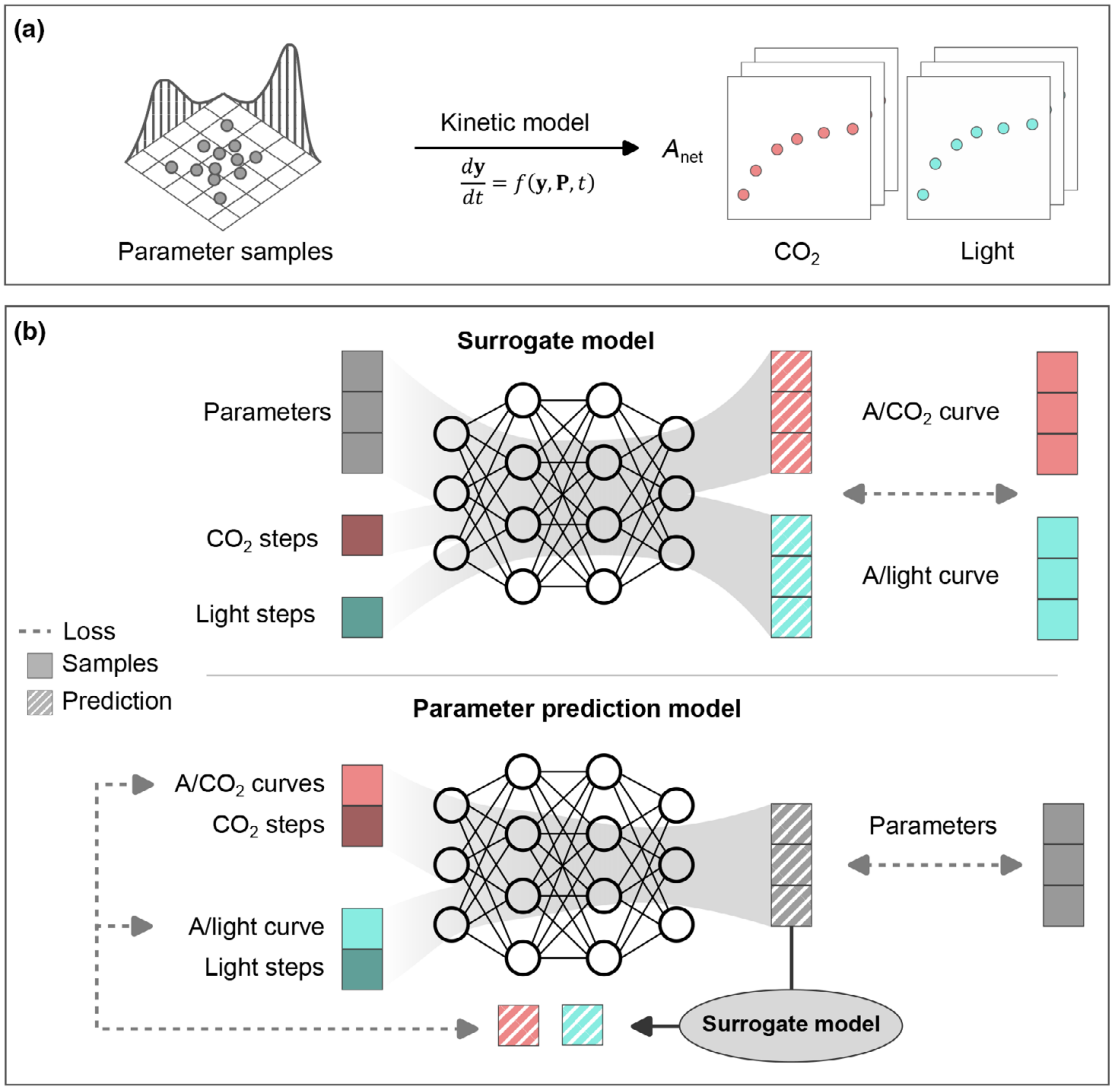
Schematic overview of the generation of artificial training data and training of neural networks in C4TUNE. (a) Based on a set of initial, feasible parameter sets, parameter samples were drawn from a multivariate log‐normal distribution (*n* = 106). For each of the sampled parameter vectors, responses of the net CO_2_ assimilation rate ($A_{\mathrm{net}}$) to different levels of ambient CO_2_ and light intensity were simulated, resulting in A/CO_2_ and A/light curves, respectively. The simulations were performed using a kinetic model of C_4_ photosynthesis (Wang *et al*., 2021), where y denotes the initial state vector of the system of ordinary differential equations (ODEs), P denotes the vector of parameters, and t is the time. (b) Inputs and outputs of the trained artificial neural networks. A surrogate model was trained to predict kinetic model simulations, that is, A/CO_2_ and A/light curves, based on a parameter vector and CO_2_ and light steps of the $A_{\mathrm{net}}$ response curves. The model weights were updated by minimizing a loss function based on the distance between the predicted and samples $A_{\mathrm{net}}$ response curves. The parameter prediction model takes A/CO_2_ and A/light curves and the associated CO_2_ and light steps as inputs and predicts a vector of kinetic model parameters. The trained surrogate model was in turn used to predict $A_{\mathrm{net}}$ response curves based on the predicted parameter vector. The loss function used to update the weights during the training process of the parameter prediction model was a weighted sum of the distance between the sampled and predicted parameter vectors and the distance between sampled curves and the curves predicted using the surrogate model.

Given that time needed for simulation of millions of response curves using the ODE solver is not feasible for this model, we trained a surrogate model that predicts $A_{\mathrm{net}}$ response curves from the CO_2_ and light intensity inputs and a set of parameters (Fig. 1b). A similar workflow involving a surrogate model has been proposed previously and has been applied to Michaelis–Menten kinetics and a *Escherichia coli* growth model (Borisyak *et al*., 2024). The main differences with the workflow by Borisyak and colleagues and the present work include the use of different error functions, as well as our consideration of the error to the associated parameter vector. Due to non‐standardized kinetic formulations in the C_4_ photosynthesis model, including algebraic equations, the convergence of the model could not be evaluated automatically. Therefore, the additional error term considering the parameter vector in the loss function was necessary because the use of the surrogate model alone was insufficient to guarantee the prediction of parameter vectors that result in feasible model simulations.

For model training and testing, the synthetic dataset (*n* = 764 804) was divided into a training set (80%) and a test set (20%).

### Surrogate model

The architecture of the surrogate model is composed of three separate input branches processing the input for CO_2_ and light response curves, as well as the parameter vector (*n* = 236). The parameter input is processed by three dense layers of dimension 128, each followed by the rectified linear unit (ReLU) activation function. The inputs relating to the experimental conditions are comprised of the steps in the CO_2_ partial pressure ($n_{CO_{2}}=11$) or light intensity ($n_{\text{light}}=6$), combined with a fixed value of light intensity and CO_2_ partial pressure for the two curves, respectively. The experimental condition inputs are each processed by a single‐layer long short‐term memory (LSTM), which considers the parameter encoding in each step. The dimension of the hidden layer in both LSTMs is 64. The encodings of experimental conditions and parameters are weighted by an attention mechanism, resulting in a joined encoding with dimension 128. The joined encoding is then combined with each of the encodings of the experimental condition, yielding two dense layers with input dimension 192 and output dimensions corresponding to the size of CO_2_ and light intensity steps, respectively. The output from both final layers is transformed by

(Eqn 7) $$40\cdot \tanh\left(x\right)+30,$$

which ensured that the range of model outputs corresponds to the criteria defined above (‘Criteria for physiologically relevant model parameterizations’ in the Materials and Methods section). A depiction of the model architecture can be found in Fig. S4.

The surrogate model inputs are *z*‐transformed. For the parameter input, the mean and SD are determined based on the training set and test set, respectively, to avoid data leakage. The means and SD of the CO_2_ partial pressures and light intensity values were calculated from the respective ranges given in the section ‘Simulations of *A* _net_ response curves’ in the Materials and Methods section. The model was trained by minimizing a loss function, which combines both the mean squared error (MSE) to the known response curves, but also punishes predictions with more than one change in monotonicity (‘Criteria for physiologically relevant model parameterizations’ in the Materials and Methods section):

(Eqn 8) $$L=L_{CO_{2}}+L_{\text{light}}$$

(Eqn 9) $$L_{CO_{2}}=m_{CO_{2}}\cdot \frac{1}{n_{CO_{2}}}\sum_{i=1}^{n_{CO_{2}}}{\left(A_{\mathrm{net}}^{\text{pred}}\left(i\right)-A_{\mathrm{net}}^{\text{sampled}}\left(i\right)\right)}^{2},$$

(Eqn 10) $$L_{\text{light}}=m_{\text{light}}\cdot \frac{1}{n_{\text{light}}}\sum_{i=1}^{n_{\text{light}}}{\left(A_{\mathrm{net}}^{\text{pred}}\left(i\right)-A_{\mathrm{net}}^{\text{sampled}}\left(i\right)\right)}^{2},$$

where L denotes the loss function, $m_{CO_{2}}$ and $m_{\text{light}}$ denote the number of changes in monotonicity in the different response curves. The numbers of CO_2_ and light steps are denoted by $n_{CO_{2}}$ and $n_{\text{light}}$. The predicted and sampled $A_{\mathrm{net}}$ values are denoted by $A_{\mathrm{net}}^{\text{pred}}$ and $A_{\mathrm{net}}^{\text{sampled}}$, respectively.

Training was performed with an initial learning rate of α=0.027, which decays linearly to 0.01α over 15 epochs. Every 15 epochs, the linear schedule was reset, but the initial learning rate was modified by a factor of γ=0.5. The model parameters were updated using stochastic gradient descend with a momentum of 0.78 and Nesterov momentum. Additionally, gradient clipping was applied with a clipping norm of 2.39. Training parameters were optimized by randomized grid search with Adaptive Successive Halving using ray tune 2.37.0 (Liaw *et al*., 2018). In this approach, multiple training runs are performed with parameter combinations randomly drawn from specified parameter distributions or choices. The process is made more efficient by stopping the training runs that perform worse than others with respect to the average loss calculated over the test set. The final model was trained over 60 epochs with a batch size of 8.

### Parameter prediction model (C4TUNE)

The neural network for parameter prediction does not predict the parameter values directly. Instead, it predicts deviations from the average parameter values ($\bar{P}$) of the training or test set, respectively. The network has two separate input branches, which take the concatenated $A_{\mathrm{net}}$ values, CO_2_ or light steps, and respective constant light intensity and CO_2_ partial pressures as inputs. These inputs, of dimensions 23 and 13, respectively, are passed to a single‐layer LSTM with a hidden layer dimension of 128. Both LSTMs are followed by a dense layer of dimension 128 and the two encodings are concatenated and passed into a dense layer of dimension 64. This combined curve encoding is then passed through three dense layers with dimensions 128, 256, and 236. The first two of these dense layers are followed by the ReLU activation function while the output of the final layer is transformed by

(Eqn 11) $$e^{\mathrm{Lx}}$$

where L is again the matrix obtained from applying Cholesky decomposition to the covariance matrix of the log‐transformed parameter values of the training or test set (as in Eqn 4). The final parameter predictions are then calculated by

(Eqn 12) $$P^{\text{pred}}=e^{\log\left(\bar{P}\right)+\mathrm{Lx}}=\bar{P}\cdot e^{\mathrm{Lx}}$$

A depiction of the model architecture can be found in Fig. S5.

The model was trained by minimizing a loss function that combines the relative error between the predicted and known parameter values, and the MSE between $A_{\mathrm{net}}$ response curves predicted using the surrogate model and the known $A_{\mathrm{net}}$ values:

(Eqn 13) $$L=\begin{array}{l} \frac{1}{n_{CO_{2}}+n_{\text{light}}}\sum_{i=1}^{n_{CO_{2}}+n_{\text{light}}}{\left(A_{\mathrm{net}}^{\text{pred}}\left(i\right)-A_{\mathrm{net}}^{\text{sampled}}\left(i\right)\right)}^{2}\\ +\;\lambda \frac{1}{n_{\text{params}}}\sum_{i=1}^{n_{\text{params}}}\frac{\left|P_{i}^{\text{pred}}-P_{i}^{\text{sampled}}\right|}{P_{i}^{\text{sampled}}}. \end{array}$$

The hyperparameter λ balances the two parts of the loss function. Here, a value of 9.12 for λ was found to yield good results. Moreover, $n_{\text{params}}$ denotes the number of predicted parameters.

This model was trained with an initial learning rate of α=0.01, and a linear decay to 0.05α over 10 epochs. After every 10 epochs, the initial learning rate was multiplied by γ=0.5. The optimization of model parameters was done using stochastic gradient descend with a momentum of 0.70. Further, gradient clipping was performed with a clipping norm of 2.48. The model architecture and training hyperparameters were tuned as described above (‘Surrogate model’ in the Materials and Methods section). The final model was trained for 60 epochs with a batch size of 8.

The neural networks were implemented using Python 3.10.14 using the PyTorch library v.2.5.1 (Ansel *et al*., 2024).

## Results

### Efficient prediction of C_4_ photosynthesis parameters in a large‐scale kinetic model

The major contribution of our work is a procedure for training an artificial neural network, C4TUNE, that predicts parameters of a large‐scale kinetic model of C_4_ photosynthesis (Wang *et al*., 2021) given gas exchange measurements. Specifically, C4TUNE makes use of the response of the net CO_2_ assimilation rate, $A_{\mathrm{net}}$, to ambient CO_2_ partial pressures and light intensities, gathered in A/CO_2_ and A/light curves, respectively, that are facile to measure (Busch *et al*., 2024). C4TUNE predicts model parameterizations that can be used to accurately simulate these experimental measurements (Fig. 1). To this end, C4TUNE was trained on a synthetic dataset of matched parameter values and A/CO_2_ and A/light curves, which was generated using the C_4_ kinetic model.

### Generation of a comprehensive synthetic dataset for neural network training

To ensure that C4TUNE can accurately predict model parameters reproducing various shapes of experimentally measured A/CO_2_ and A/light curves, the dataset used for model training must include diverse curve shapes alongside corresponding parameterizations. However, such matched experimental data on $A_{\mathrm{net}}$ response curves and model parameters are currently not available. Therefore, we generated synthetic training data by sampling values for each of the 236 tunable parameters (Dataset S1). We then used the kinetic model to simulate A/CO_2_ and A/light curves with the sampled parameter values, thus avoiding the need for matched experimental measurements (Fig. 1a). Whilst the parameter sampling can be performed at random, we opted to make use of genotype‐specific parameter values. These were obtained from a Monte‐Carlo parameter estimation strategy with data from 68 maize accessions from gas exchange experiments conducted in 2022 and 2023 (*n* = 136, see ‘Materials and Methods’ section, Xu *et al*., 2025). Whilst the estimation of the 236 parameters is not statistically acceptable due to the comparatively low number of measured data points, these estimates provide feasible model parameterizations for the measured $A_{\mathrm{net}}$ response curves. In addition, the parameters result in simulated A/CO_2_ and A/light curves of low MSE with a median of 2.38 with respect to the measurements.

**Fig. 2:**
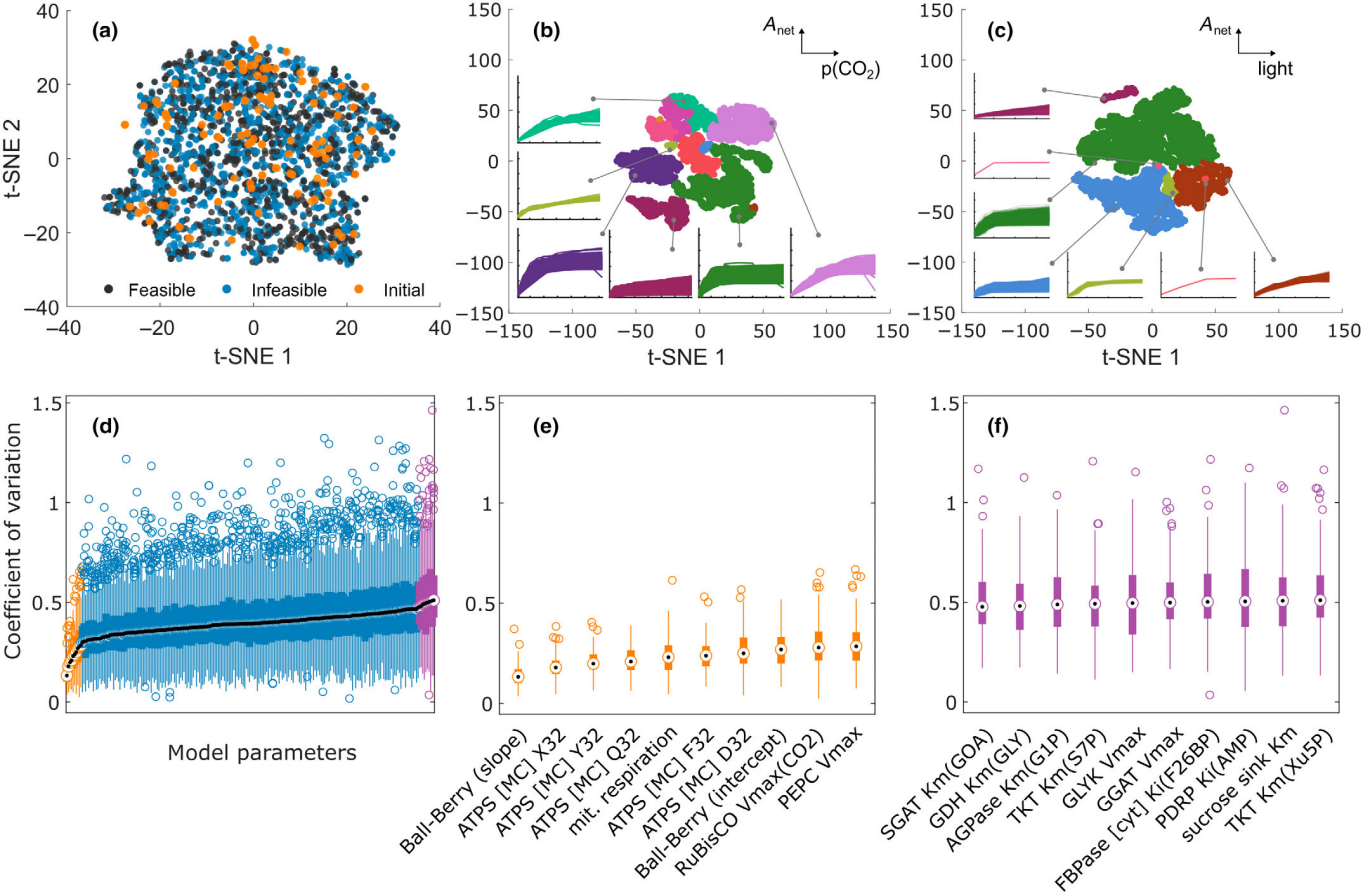
Analysis of the parameter sampling and simulation of response curves. (a) Non‐linear embedding of the sampled parameter vectors using t‐SNE (van der Maaten & Hinton, 2008). The embedding was performed on a set composed of randomly selected feasible (*n* = 1000, black) and infeasible (*n* = 1000, blue) as well as the initial parameter sets that were used to estimate the covariance structure (*n* = 136, orange). A parameter vector is feasible if the resulting model can be simulated and the resulting $A_{\mathrm{net}}$ response curves are biologically relevant; otherwise, a parameter vector is considered infeasible (see the ‘Materials and Methods’ section). (b, c) Non‐linear embedding of simulated $A_{\mathrm{net}}$ response curves to CO_2_ (b) and light (c) using t‐SNE (*n* = 10 000). To obtain clusters of different curve types, spectral embedding was performed based on the t‐SNE embedding, followed by K‐medoids clustering of the results (*K* = 12 for A/CO_2_ and *K* = 7 for A/light). A full representation of the curves in each cluster is shown in Supporting Information Figs S2 and S3. (d) Distributions of coefficients of variation (CVs) of parameters associated with highly similar A/CO_2_ and A/light curves. A random sample parameter vectors was selected (*n* = 10 000) and the associated $A_{\mathrm{net}}$ response curves were compared with all other curves in the entire dataset to find clusters of similar curves. The CV per parameter was calculated for each cluster that contained at least five members (*n* = 152). (e, f) The ten parameters with the lowest (orange, e) and highest median CV (purple, f) in ascending order, corresponding to the color code in (d). The central dot and boxes in the box charts in (d) through (f) indicate the median and 25^th^ and 75^th^ percentiles, respectively. Outlier values (circles outside whisker range) are more than 1.5× the interquartile range away from the top or bottom of the box, and whiskers connect the lower or upper quartiles with the non‐outlier minimum or maximum. AGPase, ADP‐glucose pyrophosphorylase; ATPS, ATP synthase (chloroplast); FBPase, fructose‐bisphosphatase; GDH, glycine cleavage system; GGAT, glycine transaminase; GLYK, D‐glycerate 3‐kinase; PDRP, PPDK regulatory protein; PEPC, phosphoenolpyruvate carboxylase; PPDK, pyruvate, phosphate dikinase; SGAT, serine – glyoxylate transaminase; TKT, transketolase; parameters of the electron transport equations (von Caemmerer, 2000; Wang *et al*., 2021) D32, ATP/electron ratio (whole chain+Q‐cycle); f, $\mathrm{abs}\cdot \left(1-f\right)$; F26BP, fructose‐2,6‐bisphosphate; F32, combined absorption, abs, and light quality factor; G1P, glucose‐1‐phosphate; GLY, glycine; GOA, glyoxylate; Ki, inhibitory constant, the specificity of the constants is indicated in parentheses; *K*_m_, Michaelis–Menten constant; MC, mesophyll cell; Q32, curvature parameter (θ) for the response of electron transport to light; S7P, sedoheptulose‐7‐phosphate; *V*_max_, maximum reaction velocity; X32, light partition coefficient; Xu5P, xylulose‐5‐phosphate; Y32, *J*_max_ partition coefficient.

Next, we sampled 1000 parameter vectors from log‐normal distributions for each parameter with mean and SD derived from the initial set of genotype‐specific estimates. From these, we found that for 287 parameter vectors, at least one $A_{\mathrm{net}}$ response curve simulation was infeasible. We note that a simulation was considered infeasible if the model ODEs could not be integrated or the resulting curve was not biologically relevant (see Materials and Methods for precise definition). To increase the probability of sampling feasible parameter vectors, we used the covariance structure of the feasible parameter vectors, determined above, to draw all subsequent random samples from a multivariate log‐normal distribution that resembles the structure of the feasible vectors. As a result, 10^6^ additional random parameter samples were drawn and used to simulate $A_{\mathrm{net}}$ response curves, out of which a smaller fraction (23.6%) were infeasible. A two‐dimensional non‐linear embedding of the parameter space, including feasible, infeasible, and initial parameter vectors, demonstrates the uniform coverage of the parameter space (Fig. 2a). This observation also emphasizes the difficulty in distinguishing feasible from infeasible parameter vectors.

In addition to ensuring sufficient sampling coverage of the parameter space, a synthetic dataset should capture the variety of A/CO_2_ and A/light curves shapes that may be obtained from measurements. To investigate whether the synthetic dataset contains distinct classes of curve shapes, we performed clustering for the generated A/CO_2_ and A/light curves separately. As a result, we found groups of distinct curve shapes, which mainly differ by the point where $A_{\mathrm{net}}$ reaches a plateau and the magnitude of the plateau (Fig. 2b,c), supporting the biological relevance of the generated dataset.

To further determine whether parameters are identifiable, that is a unique value corresponds to a single pair of A/CO_2_ and A/light curves, we determined the CV per parameter across identical curves. A curve was termed identical if the average distance of $A_{\mathrm{net}}$ across all CO_2_ and light steps were below a defined threshold, which was chosen well below the measurement error (see Materials and Methods). We found that the median CV between identical curves ranged between 0.13 and 0.51 with an average of 0.39 over the 236 parameters (Fig. 2d). For instance, the Michaelis–Menten constant of RuBisCO for CO_2_ (median CV = 0.36) showed values between 0.005 and 0.017 for a cluster of five identical curves (Fig. S6). Overall, the parameters with the lowest median CVs were related to model inputs, for example the Ball‐Berry model (Ball *et al*., 1987), parameters related to photosynthetic electron transport rate (e.g. X32, Y32, Q32, F32, and D32), mitochondrial respiration, and the *V*_max_ values of the RuBisCO carboxylation activity and PEP carboxylase (Fig. 2e). By contrast, parameters with the highest median CV values were related to enzymes in the Calvin‐Benson‐Bassham (CBB) cycle (transketolase, TKT), sucrose synthesis and sink (sucrose sink reaction, fructose‐bisphosphatase), C_4_ photosynthetic carbon assimilation cycle (pyruvate, phosphate dikinase (PPDK) regulatory protein), photorespiration (glycine transaminase, D‐glycerate 3‐kinase (GLYK), glycine cleavage system, serine‐glyoxylate transaminase), and starch synthesis (ADP‐glucose pyrophosphorylase) (Fig. 2f). This is in line with the expectation that parameters related to processes that integrate model inputs are more likely to affect $A_{\mathrm{net}}$ than those of downstream processes. Moreover, parameters with low CV are among the most influential parameters that discriminate feasible from infeasible parameter sets, which is expected to result in lower CVs in the synthetic dataset, which only comprises feasible parameter sets (Notes S2; Figs S7–S9). Taken together, we conclude that the parameters of the model used are not mathematically identifiable in a strict sense; that is, different parameter sets can result in identical simulation results in terms of the considered A/CO_2_ and A/light curves, within experimental error. However, the maximum CV between identical curves is below 1 for 75% of the parameters, indicating small differences between the values for each parameter that result in identical curves that warrant their prediction from data.

### Neural network as a surrogate for kinetic model simulations

The limited parameter identifiability represents a challenge in training a model to predict parameter values. To address this issue, we included the MSE between the measured response curve and the curve simulated with the predicted parameters as an additional term in the loss function for training C4TUNE. However, such a loss function requires millions of kinetic model simulations for corresponding parameterization during the training process. This is practically infeasible given that each model simulation to steady state takes at least 1.3 s per curve pair. To remedy this problem, we trained a neural network that predicts the simulation results of the kinetic model; therefore, this neural network acts as a surrogate for the kinetic model simulation. A similar idea underpins a previous study that predicts parameters of Michaelis–Menten kinetics in a model of *E. coli*'s metabolism (Borisyak *et al*., 2024). In brief, the developed surrogate model processes model parameters together with CO_2_ and light steps as inputs and predicts an A/CO_2_ and an A/light curve as output (Figs 1, S4).

**Fig. 3:**
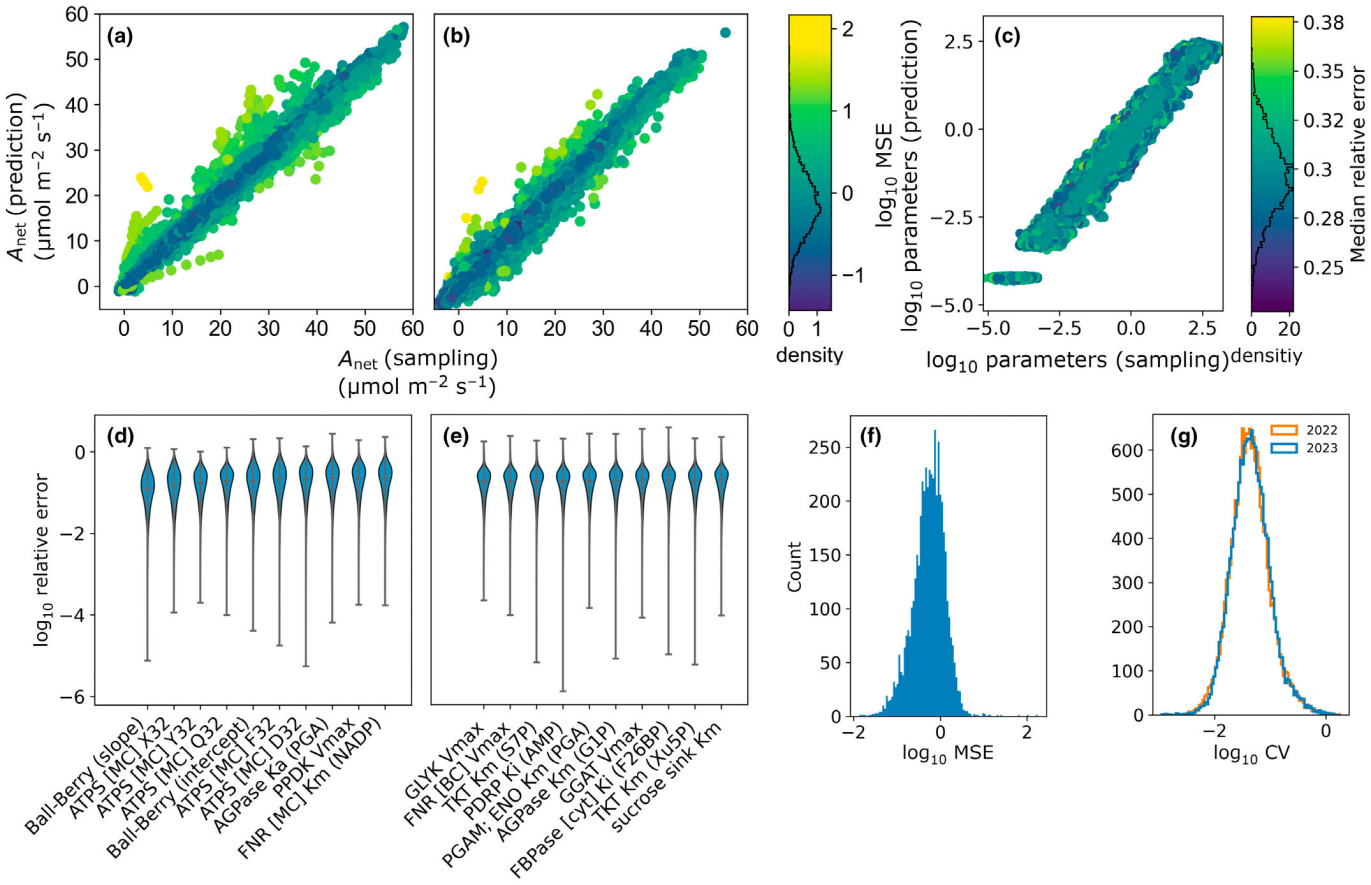
Performance of the trained surrogate and parameter prediction models. (a, b) Sampled and predicted $A_{\mathrm{net}}$ response curves to CO_2_ (a) and light steps (b) using the trained surrogate model, based on 10 000 randomly selected parameter vectors from an unseen test set. The color gradient indicates the average mean squared error (MSE) across both curve types. The black line in the colorbar shows the distribution of the logarithmic average MSE values. (c) Sampled and predicted parameter values using the trained parameter prediction model (*n* = 5000). The color gradient indicates the average relative error per parameter vector. The black line in the colorbar shows the distribution of median relative errors. (d, e) Distributions of logarithmic relative errors of the 10 parameters with the lowest (d) and highest median relative errors (e) in ascending order. The whiskers extend to the lowest and highest values, respectively, and the vertical line within the violins represents the median value. (f) Distribution of logarithmic MSE values of kinetic model simulations using the predicted parameters (*n* = 5000), compared with the curves simulated with the corresponding parameter samples. The MSE values are the average MSE values of both curve types. (g) Logarithmic coefficients of variation (CV) of predicted parameters for 68 maize accession and year grown in 2022 and 2023, respectively. For each accession and year, 10 A/CO_2_ and A/light curves were randomly generated within the respective experimental error and the associated parameters were predicted using C4TUNE. AGPase, ADP‐glucose pyrophosphorylase; ATPS, ATP‐synthase (chloroplast); ENO, 2‐phosphoglycerate enolase; FBPase, fructose‐bisphosphatase; FNR, ferredoxin – NADP^+^ reductase; GGAT, glycine transaminase; GLYK, D‐glycerate 3‐kinase; PDRP, PPDK regulatory protein; PGAM, phosphoglycerate mutase; phosphate dikinase; PPDK, pyruvate; TKT, transketolase; parameters of the electron transport equations (von Caemmerer, 2000; Wang *et al*., 2021) D32, ATP/electron ratio (whole chain+Q‐cycle); f, $\mathrm{abs}\cdot \left(1-f\right)$; F26BP, fructose‐2,6‐bisphosphate; F32, combined absorption, abs, and light quality factor; G1P, glucose‐1‐phosphate; Ki, inhibitory constant; *K*_m_, Michaelis–Menten constant; MC, mesophyll cell; PGA, 3‐phosphoglycerate; Q32, curvature parameter (θ) for the response of electron transport to light; S7P, sedoheptulose‐7‐phosphate; *V*_max_, maximum reaction velocity, the specificity of the constants is indicated in parentheses; X32, light partition coefficient; Xu5P, xylulose‐5‐phosphate; Y32, *J*_max_ partition coefficient.

Following model training, the performance of the surrogate model was assessed on an unseen test set (Fig. 3a,b). The median MSE was 0.75 ± 0.40 (median ± median absolute deviation (MAD)) with 95% of the MSE values below 3.55. The performance was slightly better for A/CO_2_ curves (Fig. 3a, MSE = 0.56 ± 0.41, *R* ^2^ = 0.99 ± 0.00, median ± MAD), compared to A/light curves (Fig. 3b, MSE = 0.84 ± 0.45, *R* ^2^ = 0.99 ± 0.01, median ± MAD). Moreover, the MSE values from A/CO_2_ and A/light curves generated with the same parameter values showed a Pearson correlation coefficient of 0.78. This indicates, as expected, that for most of the parameter vectors, there is dependence between the prediction qualities for both curve types. An investigation of critical parameters for the surrogate model performance revealed an important role of the *V*_max_ value of the chloroplastic transketolase (Notes S3; Fig. S10). Along with *K*_M_ values associated with transketolase, these parameters also showed high CV values among identical curves, indicating a correspondence between identifiability and surrogate model performance. Overall, the surrogate model predictions showed a very high similarity to the curves simulated by the kinetic model, showing that the model can safely be used to replace the model ODEs for the purpose of curve prediction in model training.

### C4TUNE efficiently predicts model parameterizations that match measured response curves

The developed deep learning model for the prediction of kinetic model parameters, C4TUNE, takes as inputs the two curve types and their respective CO_2_ and light steps, and predicts deviations from the average parameter values as final output (Fig. S5; the Materials and Methods). The performance of the final, trained model was assessed using a random subsample of the unseen test set comprising 5000 samples, resulting in a median relative error between true and predicted parameter values of 0.30 ± 0.16 (median ± MAD, Fig. 3c). A closer inspection of the distribution of relative errors of predicted parameters revealed that the median relative errors across the test samples showed differences across the parameters with the highest median relative error of 0.40 ± 0.20 (median ± MAD) observed for the *K*_M_ value of the sucrose sink reaction for sucrose and the lowest median relative error of 0.13 ± 0.07 observed for the slope of the Ball‐Berry model. In agreement with the observed CV values of the parameters across identical curves (Pearson *r* = 0.96), the parameters with the highest and lowest relative errors largely overlapped with the parameters with high and low CV, respectively (Fig. 3d,e). This shows that the predictability of the parameters is directly related to their unique association with distinct curve shapes, influencing the capacity of the neural network to learn the relationship between parameter values and associated $A_{\mathrm{net}}$ response curves.

Further, the median MSE between true A/CO_2_ and A/light curves and the curves predicted by the surrogate model was 0.06 ± 0.03 (median ± MAD). Notably, this error is more than tenfold lower than the MSE of the surrogate model with sampled parameters as input. On one hand, this shows that the predicted parameters result in surrogate model curve predictions which are practically indistinguishable from the sample curves. On the other hand, it may also indicate that C4TUNE is overfitted to predict parameters that result in exceptional curve predictions with the surrogate model, but not necessarily when using them to integrate the model ODEs. Given that the surrogate model does not discriminate between feasible and infeasible parameter sets, the low MSE between predicted and true curves for the parameter prediction model does not guarantee that C4TUNE results in feasible parameter vectors for kinetic model simulation. To test whether the predicted parameters can be used to simulate the kinetic model with a similar performance to the surrogate model, we re‐ran the curve simulations using the kinetic model with 5000 randomly selected predicted parameter vectors. We found that over 99% of the predicted parameter vectors resulted in successful simulations, with a median MSE of 0.58 ± 0.30 (median ± MAD) with 95% of the MSE values lower than 1.86 (Fig. 3f). Here, too, the median MSE for A/light curves (0.64 ± 0.29) was higher than for A/CO_2_ curves (0.48 ± 0.29). Importantly, most predicted parameters could be used in simulations using the original kinetic model with a low error, showing that C4TUNE is not overfit to the surrogate model.

To demonstrate that the predicted parameters correspond to estimates derived from experimental data, we applied C4TUNE to a recent gas exchange measurement for maize and *Sorghum bicolor* published by Almeida *et al*., 2025 (Notes S4). Notably, a different constant irradiance (2000 μmol m^−2^ s^−1^) was used to measure A/CO_2_ curves in this study, compared to an irradiance of 1800 μmol m^−2^ s^−1^, which was used to train C4TUNE. First, we predicted the model parameters and assessed the prediction quality by simulating the *A*_net_ response curves using the kinetic model. Across all genotypes and conditions, the median MSE between measured and predicted *A*_net_ values was 7.1. Second, we compared values for $V_{c_{\max}}$ and $V_{p_{\max}}$ between the estimations by Almeida *et al*., 2025 and our predictions, and found Pearson correlations of *r* = 0.86 and *r* = 0.48, respectively (Fig. S11).

Taken together, the presented parameter prediction model showed solid performance in recovering kinetic model parameters underlying a diverse set of simulated $A_{\mathrm{net}}$ response curves. The results demonstrated the applicability of the approach to produce results that can be used for downstream analysis, such as additional curve simulations for unseen conditions or simulation of mutant phenotypes. Moreover, we demonstrated that the predicted values from C4TUNE and estimates from classical photosynthesis models correspond in magnitude and show high correlation. The limited identifiability of the parameters with respect to the considered A/CO_2_ and A/light curves was reflected in a lower bound on the relative errors between predicted and true parameter values. We expect that a large proportion of the limited identifiability and resulting reduction in predictability is caused by compensatory effect between parameters, especially *V*_max_ and *K*_M_ values of the same enzyme. To identify compensatory effects between parameters, we scanned the sets of identical curves determined earlier for parameter pairs, which are correlated to each other and to *A*_net_, and show a compensatory effect (Notes S5). The parameter pairs were ranked by considering both kinds of correlations, and 14 pairs were identified. The top 3 pairs with the highest compensation effects were (1) the *K*_i_ value for *P*_i_ and the *K*_M'_ value for ribulose‐5‐phosphate of phosphoribulokinase (PRK), (2) the *V*_max_ value for phosphoglycerate kinase and glyceraldehyde 3‐phosphate dehydrogenase and the light regulation constant for RuBisCO, and (3) the *K*_M_ value for HCO_3_ and the *V*_max_ value of phosphoenolpyruvate carboxylase (PEPC) (Table S1). Notably, compensation effects were not observed uniformly across all sets of identical curves and *A*_net_ values for the different pairs, indicating that these effects depend on the values of other parameters and environmental conditions. The identifiability of some of the parameters could likely be improved by including additional experiments, for example gas exchange measurements under different environmental conditions, protein abundances, or metabolite concentrations.

### C4TUNE provides precise maize genotype‐specific model parameters that result in low simulation error

To evaluate the performance of C4TUNE when applied to experimental measurements, we predicted parameters for 68 maize accessions grown in 2022 and 2023. To verify that the predicted parameters are indeed relevant, we performed simulations for $A_{\mathrm{net}}$ response curves with the surrogate and kinetic model for both growing seasons, respectively. The median MSE values between the experimental measurements and surrogate model predictions were 2.26 (2022) and 1.25 (2023). For the kinetic model simulations, the median MSE values were 2.38 and 1.43 for 2022 and 2023, respectively. This result shows that, while the measured A/CO_2_ and A/light curve pairs were not considered alongside the training response curves generated by the kinetic model, C4TUNE was still able to predict parameters that resulted in curves closely resembling the experimental observations (Fig. S12).

The results presented above were obtained by predicting parameter vectors for average values of $A_{\mathrm{net}}$ response curves over the biological replicates. However, there can be considerable variation across replicates, with median CV values of 0.14 ± 0.05 (2022) and 0.16 ± 0.06 (2023) for A/CO_2_ curves and 0.20 ± 0.10 (2022) and 0.23 ± 0.10 (2023) for A/light curves. To investigate how the uncertainty in experimental measurements is reflected in the predicted parameters, we sampled 10 curves from a normal distribution with mean and SD specific to the measurement for each accession and year. Next, we used C4TUNE to predict parameters which were in turn used for simulation with the surrogate model and the kinetic model. The resulting curves showed median MSE values of 3.09 for 2022 and 5.33 for 2023 using the surrogate model (median $R^{2}=0.98$ and 0.97), and 4.73 and 7.95, respectively, using the kinetic model (median $R^{2}=0.98$ and 0.95, Fig. S13). The median CV of the predicted parameters was 0.04 for both years (Fig. 3g), demonstrating that C4TUNE can reliably predict parameters for perturbed curve inputs, sampled within the experimental error. These values were almost tenfold lower compared to the CVs of parameters associated with identical curves, despite the higher variation in the synthetic response curves used for training. This observation showed that the prediction performance of C4TUNE is robust against experimental uncertainty in the input data. It further indicated that the parameter identifiability is higher based on the maize‐specific $A_{\mathrm{net}}$ response curves. Given the higher CV of parameters associated with identical curves in the dataset, this also means that C4TUNE is less likely to include extreme values of parameter values that result in equally accurate curve simulations.

### Predicted model parameters pinpoint photosynthetic limitations

**Fig. 4:**
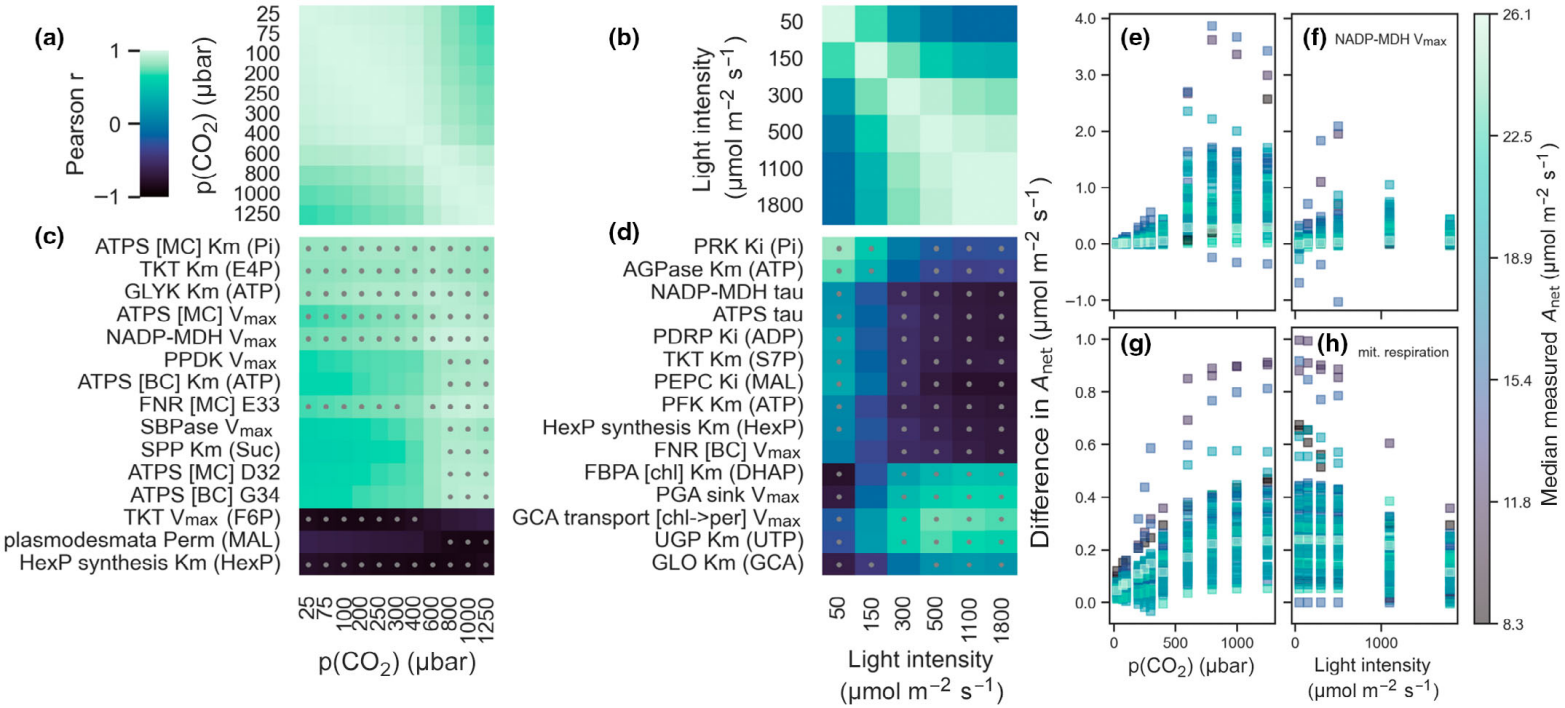
Correlations between $A_{\mathrm{net}}$ and parameter values. Pairwise Pearson correlations between (a, b) $A_{\mathrm{net}}$ values at different CO_2_ and light steps and (c, d) between $A_{\mathrm{net}}$ values and log‐transformed parameter values were determined. The $A_{\mathrm{net}}$ response curves (A/CO_2_ and A/light curves) were obtained from 68 maize genotypes grown in 2022. The heatmaps in (c) and (d) show the correlations of the 15 parameters that ranked highest when sorted by the median correlation across all CO_2_ or light steps, respectively. The color code indicating correlation is the same across all panels, as indicated by the common color bar. Gray dots in the heatmap tiles indicate that the respective correlation coefficient is higher than the minimum of pairwise correlation between *A*_net_ values measured at the different ambient CO_2_ or light intensity levels, respectively (shown in a and b). Parameters that showed the highest sign‐consistent correlations to A/CO_2_ and A/light were considered as potential targets for improvement of photosynthetic efficiency. Their values were updated individually to the highest or lowest value of the parameter predicted across the entire population, depending on the sign of the correlation. The updated parameter sets were used to simulate A/CO_2_ and A/light curves using the kinetic model and the differences to the original curve were quantified for all 68 genotypes. The simulated curves with the highest median differences resulted from increased *V*_max_ values of the NADP‐MDH enzyme, which showed strong correlations to A/CO_2_ curves (e, f), and from reduced mitochondrial respiration, which showed a strong correlation to A/light curves (g, h). The colors in (e–h) indicate the median $A_{\mathrm{net}}$ for each curve type per accession, as shown in the colorbar. AGPase, ADP‐glucose pyrophosphorylase; ATPS, ATP synthase; chl, chloroplast; D32, ATP/electron ratio (whole chain+Q‐cycle); DHAP, dihydroxyacetone‐phosphate; E33, NADPH/electron ratio (whole chain+Q‐cycle); E4P, erythrose‐4‐phosphate; F6P, fructose‐6‐phosphate; FBPA, fructose‐bisphosphate aldolase; FNR, ferredoxin – NADP+ reductase; G1P, glucose‐1‐phosphate; G34, ATP/electron ratio (whole chain); GAP, glyceraldehyde‐3‐phosphate; GCA, glycolate; GLO, glycolate oxidase; GLYK, D‐glycerate 3‐kinase; HexP, hexose phoshates; *J*_max_, maximum rate of photosynthetic electron transport; *K*_m_, Michaelis–Menten constant; MAL, malate; MEP, proton:pyruvate cotransporter; NADPH‐MDH, malate dehydrogenase; PDRP, PPDK regulatory protein; PEPC, phosphoenolpyruvate carboxylase; per, peroxisome; Perm, permeability; PFK, 6‐phosphofructo‐2‐kinase; PGA, 3‐phosphoglycerate; phosphate dikinase; PPDK, pyruvate; PPDK_I, inactive PPDK; PRK, phosphoribulokinase; PYR, pyruvate; RuBisCO, ribulose‐1,5‐bisphosphate carboxylase/oxygenase; S7P, sedoheptulose‐7‐phosphate; SBPase, sedoheptulose‐bisphosphatase; SPP, sucrose‐phosphate phosphatase; Suc, sucrose; tau, rate constant of light‐regulated enzyme activation; TKT, transketolase; UGP, UTP – glucose‐1‐phosphate uridylyltransferase; *V*_max_, maximal reaction rate.

Next, we used the accession‐specific model parameterizations for the 2022 growing season to identify parameters that are highly correlated with changes in net assimilation rate. To this end, we determined the pairwise correlations between the predicted parameters and $A_{\mathrm{net}}$ values at each CO_2_ or light intensity step. In addition, pairwise Pearson correlations, r, between $A_{\mathrm{net}}$ values at different steps of the A/CO_2_ and A/light curves were calculated to assess their agreement. We found that the $A_{\mathrm{net}}$ values at the different CO_2_ levels were highly correlated with each other ($0.68\leq \min\left(\left|r\right|\right)\leq 0.88$), with pronounced, strong correlations between 25 and 400 μbar and from 600 to 1250 μbar (Fig. 4a). For the A/light curves, $A_{\mathrm{net}}$ values measured at high light intensities were highly correlated across the genotypes, while there were low and even partly negative correlations between $A_{\mathrm{net}}$ values measured at low and high light intensities ($0.01\leq \min\left(\left|r\right|\right)\leq 0.36$, Fig. 4b), indicating pronounced genotype‐by‐environment interactions for different CO_2_ inputs (as environments). Given that the model structure and initial concentrations remained the same across genotypes, the photosynthesis parameters are expected to determine the observed dependencies between the steps of the $A_{\mathrm{net}}$ response curves as they allow the ODE simulations to match genotype‐specific curve pairs. Therefore, the minimum absolute correlations per CO_2_ or light step between the measured $A_{\mathrm{net}}$ values were used as thresholds to determine representative associations between $A_{\mathrm{net}}$ response curves and model parameters.

We found that some of the parameters whose values showed a strong association with $A_{\mathrm{net}}$ also exhibited sign‐consistent correlations across the CO_2_ and light steps of the two curve types (Fig. 4c). Moreover, we found that the correlation of the parameters with $A_{\mathrm{net}}$ was dependent on the CO_2_ or light level for most of the parameters. For instance, the permeability of plasmodesmata for malate or the *V*_max_ value of PPDK only showed high correlations with $A_{\mathrm{net}}$ at high ambient CO_2_ partial pressures. This trend was even stronger for the parameters with the highest correlations with A/light curves (Fig. 4d). Here, we also identified parameters where correlations with $A_{\mathrm{net}}$ changed signs across different light intensities, that may be relevant in a canopy setting (Slattery & Ort, 2021). For instance, this was observed for the *K*_M_ value of the chloroplastic fructose‐bisphosphate aldolase for dihydroxyacetone‐phosphate, which suggests that increasing its value leads to a higher photosynthetic efficiency at high light intensities, whilst resulting in a decreased efficiency at low light intensity. The opposite effect was observed for the *K*_i_ value of PEPC for malate: a high value (low inhibitory effect) would be beneficial to photosynthesis at low light intensity, but tends to lead to lower $A_{\mathrm{net}}$ at high light intensities, which is relevant with regards to ongoing work to elucidate PEPC structure and function (DiMario *et al*., 2021, 2023; Carvalho *et al*., 2023). However, parameters showing such ambivalent effects may not be the most suitable targets for increasing photosynthetic efficiency. Therefore, we focused only on parameters exhibiting sign‐consistent correlations.

To this end, the obtained correlations were filtered for sign‐consistency and magnitude using the thresholds for minimum pairwise correlations determined above. For the response of $A_{\mathrm{net}}$ to ambient CO_2_, we found highly correlated parameters associated with the CBB cycle (*K*_M_ value of transketolase for erythrose 4‐phosphate (E4P)), photorespiration (*K*_M_ values of glycerate kinase for ATP), light reactions (*V*_max_ and *K*_M_ of the chloroplastic ATP synthase for phosphate), C_4_ photosynthetic carbon assimilation cycle (*V*_max_ of NADP malate dehydrogenase, NADP‐MDH), and the generation of hexose phosphates. The parameters with the highest median correlations pertained to reaction steps involved in mitochondrial respiration, C_4_ photosynthetic carbon assimilation cycle, light reactions, transport of C_4_ carboxylic acid and triose‐phosphate, photorespiration, and sucrose synthesis (Dataset S2). These parameters present possible targets for alleviating limitations on photosynthetic efficiency, due to their consistent and strong associations with $A_{\mathrm{net}}$ over multiple CO_2_ partial pressures and light intensities for the considered maize accessions. To assess, whether these limitations can also be found in other genotypes or species, we have analyzed the measurements by Almeida *et al*., 2025 following the same correlation‐based procedure to identify limiting factors. The results showed that the sets of relevant targets overlap with the results described above, however some factors were only exclusively found on either of the two datasets (Figs S14, S15).

To estimate the potential impact of optimizing the identified parameters to improve photosynthetic efficiency, we individually modified their values to the respective most extreme value predicted for the population in 2022. These were then used to parameterize the kinetic model and to simulate updated A/CO_2_ and A/light curves. We observed that most of the single parameter changes only resulted in small changes in $A_{\mathrm{net}}$ (Dataset S2). However, we found that an increase in the *V*_max_ of NADP malate dehydrogenase was predicted to result in $A_{\mathrm{net}}$ changes between −1.03 and 3.87 μmol m^−2^ s^−1^ (−89% and + 19.8%) across all genotypes and simulated conditions with a median increase of 0.08 μmol m^−2^ s^−1^ (+0.53%, Fig. 4e,f; Dataset S2). Similarly, our predictions indicated that a decrease in mitochondrial respiration rate, a fixed value that increases cytosolic CO_2_ concentrations in both cell types resulted in changes of $A_{\mathrm{net}}$ in the range from −0.03 to 1.00 μmol m^−2^ s^−1^ (−0.05% and + 1477%, Fig. 4g,h) with a median increase of 0.14 μmol m^−2^ s^−1^ (0.72%). The changes in $A_{\mathrm{net}}$ were strongest at high CO_2_ levels and low to medium light intensities. Moreover, the parameter changes had the strongest impact on accessions with overall low to medium $A_{\mathrm{net}}$ values (see Figs S16–S18 for example curves). Notably, the parameter values were optimized using extreme values within the predicted genotype‐specific parameters, while the corresponding extreme parameter values in the entire generated dataset were mostly considerably higher or lower (Dataset S2). Although these could yield greater increases in simulated photosynthetic rates (Fig. S19), we confined our optimization to the genotype‐specific predictions to prioritize the biological relevance of the proposed changes. As a control, we also simulated the increase of RuBisCO content, which has previously been shown to increase light‐saturated $A_{\mathrm{net}}$ by 15% in maize (Salesse‐Smith *et al*., 2018). In these simulations, we increased the *V*_max_ values of the RuBisCO carboxylation and oxygenation reactions to the respective maximum of the predicted values for this parameter in the employed maize population. As a result, we found that the median increase in the simulated $A_{\mathrm{net}}$ values across all genotypes was in the range from 0.4 to 5.6% (Fig. S20). When the maximum values from the artificial dataset were used instead, the increases ranged between −2.3 and 3.4%.

In addition to identifying individual limiting factors, the predicted parameters can be used to determine pairs of co‐limiting parameters. Briefly, parameter pairs were considered co‐limiting if they can only explain variance in *A*_net_ well jointly, but not individually (Notes S6). As a result, we found 277 pairs of co‐limiting parameter pairs for the 2022 growing season (Table S2). The three parameter pairs with the strongest improvement between the individual and pairwise explanations of *A*_net_, which related to enzymatic reactions, were the *K*_M_ value of PEPC in combination with the *K*_M_ value of GLYK for ATP or the *V*_max_ value of NADP‐MDH, respectively, and the *K*_i_ value of cytosolic fructose‐bisphosphatase for *P*_i_ and the *K*_i_ value of fructose‐2,6‐bisphosphatase for *P*_i_.

These results demonstrate that C4TUNE applied to data sets from a population of genotypes can pinpoint enzyme parameters that limit photosynthetic efficiency by performing simple correlation analysis. Furthermore, the correlations across the full range of the A/CO_2_ and A/light curves reveal the impact of the parameter change under different environmental conditions, which could partly be confirmed by model simulations. Therefore, C4TUNE can contribute to the discovery of novel targets for alleviating limitations on photosynthetic efficiency using precise parameter predictions.

## Discussion

Here we present C4TUNE, a deep learning framework for the prediction of C_4_ photosynthesis parameters from gas exchange measurements. The model takes as inputs the environment parameters and $A_{\mathrm{net}}$ measurement of A/CO_2_ and A/light curves and predicts a genotype‐specific set of kinetic model parameters (Wang *et al*., 2021). We trained C4TUNE on a large synthetic dataset generated by kinetic model simulations from randomly sampled model parameterizations. Using a deep learning architecture to predict model parameters provides multiple advantages over classical approaches: (1) the non‐linearities of the kinetic model are automatically captured by non‐linear activations, (2) additional constraints can be easily integrated into the model and training process, (3) very large datasets can be processed efficiently, and (4) the number of predicted parameters is not limited by the number of measurements as in statistical estimation relying on measures like $\chi^{2}$ values. For instance, we integrated the known covariance structure of the parameters to transform predicted deviations from average parameter values. To consider the limited identifiability of the model parameters, a second neural network (i.e. surrogate model) was trained to predict A/CO_2_ and A/light curve pairs from environmental parameters and kinetic model parameters. This allowed the consideration of parameter simulation errors directly and efficiently during model training. Notably, the known biologically meaningful ranges of $A_{\mathrm{net}}$ values as well as constraints on the shapes of predicted simulated A/CO_2_ and A/light curves were integrated via the surrogate model architecture and training. By allowing the prediction of full model parameterizations from A/CO_2_ and A/light curves, C4TUNE increases the number of predicted parameters, compared to previous studies (Song *et al*., 2017; Kannan *et al*., 2019; Zhao *et al*., 2021; Shameer *et al*., 2022; He *et al*., 2024; Vijayakumar *et al*., 2024), facilitating the use of kinetic photosynthesis models in different genotypes.

While neural networks have already been used for kinetic model parameterization (Yazdani *et al*., 2020; Choudhury *et al*., 2022, 2024; Borisyak *et al*., 2024), their application with existing photosynthesis models is not straightforward. The challenges include a high number of parameters and the use of non‐standard enzyme kinetics and algebraic equations in the ODEs, which hamper the application of the existing neural‐network‐based approaches. To address these issues, C4TUNE relies on a surrogate model to evaluate whether the predicted parameters can be used to obtain meaningful model simulations. Whilst the use of a surrogate model has been proposed previously (Borisyak *et al*., 2024), here we advanced on this work by also considering the parameter prediction error to train C4TUNE. Importantly, the use of these two neural networks enables parameter prediction independent of the nature of enzyme kinetic formulations used in the kinetic model, a strategy that can be easily transferred to predict parameters in different large‐scale kinetic models.

After model training, the surrogate model achieved a median MSE of 0.75, while classical machine learning approaches like a K‐nearest neighbors (K‐NN) regression model (*K* = 100) and a random forest regression achieved median MSE values of 18.61 and 8.95 (Fig. S21A). The trained C4TUNE model predicted model parameters with a median relative error of 0.30 (Fig. S21B). While a similar relative error could be achieved by a K‐NN (*K* = 100, relative error: 0.30) and a random forest regression model (relative error: 0.30), importantly, parameter predictions from each of these approaches gave rise to high errors in the kinetic model simulations. Here, the parameters predicted by C4TUNE resulted in a median MSE of 0.58 (median $R^{2}=0.94$), while the K‐NN and random forest models predictions resulted in two‐orders of magnitude higher median MSE values of 72.81 and 84.66 (median $R^{2}=0.31$ and 0.26), respectively (*n* = 100, Fig. S21C,D). These results demonstrate the proposed deep learning approach learned to predict precise and biologically relevant parameter sets, which was not possible using classical machine learning approaches.

C4TUNE was trained to predict parameters using a specific experimental setup. We assumed the availability of both A/CO_2_ and A/light curves, measured at specific ambient CO_2_ partial pressures and light intensities as well as different constant light intensities and CO_2_ levels, respectively. While $A_{\mathrm{net}}$ responses to different CO_2_ and light steps within each response curve could be accommodated by imputing $A_{\mathrm{net}}$ values at the currently used steps from a curve fit, the model training would have to be repeated if the curves were measured at different constant light or CO_2_ levels. This includes re‐running the automated parameter sampling and either retraining the model from the beginning or starting the training process from the current weights and biases. The sampling process is the most critical step, which can be completed within 12–24 h, depending on the level of parallelization. The subsequent model training takes less than 24 h using a standard laptop computer, which is expected to be strongly reduced when the training is started from the current model parameters.

To demonstrate the use of C4TUNE with experimental data, we used it to predict parameters from gas exchange measurements for a population of 68 genotypes from a maize MAGIC population. To assess the quality of the parameter predictions, they were used to predict and simulate the same A/CO_2_ and A/light curves with the surrogate and the kinetic model. Ideally, the obtained curve simulations closely match the input curves, indicating that an appropriate model parameterization was identified. Indeed, the obtained curves showed median MSE values of 2.26 and 1.25 using the surrogate model and 2.38 and 1.43 using the kinetic model for 2022 and 2023, respectively. This demonstrates that C4TUNE is able to predict parameters for the model of C_4_ photosynthesis by Wang *et al*., 2021, which result in simulations that closely match the experimental measurements. By correlating the predicted genotype‐specific parameter vectors to the measured curve pairs, we identified parameters that limit photosynthesis under the considered environmental conditions. Notably, C4TUNE enables the prediction of all 236 tunable model parameters, vastly increasing the number of parameter predictions and estimations compared to previous studies (Song *et al*., 2017; Kannan *et al*., 2019; Zhao *et al*., 2021; Shameer *et al*., 2022; He *et al*., 2024; Vijayakumar *et al*., 2024). Among these, we identified a set of parameters that showed consistently high correlations with $A_{\mathrm{net}}$ across both curve types, which present potential targets for improving photosynthetic efficiency in the considered accessions. Several of these targets have been described in a previous study (Sales *et al*., 2021), including PEPC, NADP‐MDH, PPDK, PRK, and sedoheptulose‐bisphosphatase (SBPase). Measurements of PEPC kinetic parameters have shown substantial variability, which further indicates its potential as a target, as shown by modeling (DiMario *et al*., 2021). While SBPase presents a promising target as shown in C_3_ plants (Simkin *et al*., 2019), increasing its expression in *Setaria viridis* did not yield the same effects on photosynthesis and growth (Ermakova *et al*., 2023, 2026). Additional targets include chloroplastic ATP synthase, which may play a limiting role due to its central role in ATP generation and has been shown to impact electron transport rate in rice and may therefore also play an influential role in C_4_ plants (Ermakova *et al*., 2022, 2026). Interestingly, we found that about half of the reactions associated with the parameters depicted in Fig. 4(c,d) involve either ATP, *P*_i_, or PP_i_, indicating possible spurious relationships associated with the ATP level in both cell types. The balance between ATP and NADPH levels may also be a confounding factor, given that a ferredoxin – NADP^+^ reductase parameter was also among the limiting factors. Moreover, results from a previous modeling study show that the ATP in mesophyll cells and bundle sheath cells may have distinct optima in relation to changes in other parameters, indicating that a tight control is needed (Zhao *et al*., 2022). The role of linear and cyclic electron transport and associated parameters in adjusting the ATP : NADPH ratio (Yamori & Shikanai, 2016; Ermakova *et al*., 2026) could be tested by adding more extensive light reactions to the model as available from other models (Zhu *et al*., 2005, 2013). Our analysis also identified TKT as a limiting factor. While the *V*_max_ value of TKT was limiting only at low CO_2_ levels, the *K*_M_ value for E4P was found to be limiting at all CO_2_ levels. Given the important role of this enzyme in the regeneration of ribulose‐1,5‐bisphosphate in the CBB cycle and partitioning between sucrose and starch synthesis pathways in C_3_ plants (Raines, 2003), TKT may exert control over carbon fixation in C_4_ photosynthesis as well. Alternatively, the TKT‐related parameters may have a balancing role, in the sense that their correlation with *A*_net_ is induced by changes in one or more other parameters, correlated to *A*_net_. Further, GLYK, which catalyzes the final step in photorespiration and produces 3PGA, was found to be across all steps of the A/CO_2_ curves. Although photorespiration only plays a minor role in C_4_ photosynthesis, it may play a role in connecting CO_2_ fixation in the bundle sheath cells and sucrose synthesis in the mesophyll cells (Kleczkowski & Igamberdiev, 2024). However, a closer inspection of the kinetic model showed that GLYK was placed in the bundle sheath cells, while it was almost exclusively expressed in mesophyll cells in C_4_ species (Usuda & Edwards, 1980). Finally, the *K*_M_ value for hexose phosphate (HexP) of an artificial reaction that simulates the generation of HexP, which is distributed across specific HexP species in each simulation step, was found to be limiting across all steps of the A/CO_2_ and almost all steps of the A/light response curve. Because the *K*_M_ value describes the affinity to the product of the reaction, an increase in its value would increase the flux through the reaction. On the contrary, the parameter was negatively correlated with *A*_net_, suggesting stronger negative feedback by product inhibition is related to higher *A*_net_ values. Additional targets, including mesophyll conductance (Ermakova *et al*., 2021) and carbonic anhydrase (Ermakova *et al*., 2026), were not identified in our analysis.

The identified targets could be partly confirmed by kinetic model simulations with optimized parameters derived from within the population of maize genotypes. The simulation results obtained by increasing the *V*_max_ values of the RuBisCO carboxylation and oxygenation reactions demonstrate that the improvement of photosynthetic efficiency by updating a single factor can be reproduced using the predicted parameters. However, the impact of increasing this parameter was not predicted to improve $A_{\mathrm{net}}$ across all genotypes and conditions equally, indicating that the impact of parameter optimizations varies even between closely related genotypes. This highlights the need for genotype‐specific model parameterizations for the identification of targets for improving photosynthetic efficiency. While this approach focuses on the identification of limiting factors under steady state conditions, limiting factors in dynamic conditions or different steady state conditions can be determined by performing simulations and sensitivity analyses using the parameterized kinetic model.

C4TUNE can be readily applied to populations of different species with C_4_ photosynthesis. This is rendered possible by the facile retraining of C4TUNE within 24–36 h for a different experimental setup or a different C_4_ subtype. For instance, C4TUNE can be used to generate genome‐specific kinetic models as additional features in genomic prediction of photosynthesis rate (Xu *et al*., 2025). Future efforts should also emphasize the need for validation of the predicted parameter values through enzymatic assays and other approaches that make use of diverse omics data (Ferreira *et al*., 2024). We expect C4TUNE to facilitate the engineering of and selection for traits linked to photosynthetic efficiency, contributing to improved photosynthetic efficiency and climate resilience of C_4_ crop species.

## Competing interests

None declared.

## Author contributions

PW contributed to the conceptualization, methodology, software development, investigation, writing of the original draft, writing of the review and editing, and visualization. JF contributed to the investigation and data curation. RX contributed to the software development and investigation. JK contributed to the conceptualization, writing of the review and editing, supervision, project administration and funding acquisition. ZN contributed to the conceptualization, methodology, writing of the review and editing, supervision, project administration and funding acquisition.

## Disclaimer

The New Phytologist Foundation remains neutral with regard to jurisdictional claims in maps and in any institutional affiliations.

## Supporting information


**Dataset S1** Descriptions of the 236 tunable parameters in the kinetic model of C_4_ photosynthesis.

**Dataset S2** Model simulation results after updating the genotype‐specific parameters for 68 maize accessions grown in 2022.

**Fig. S1** Clustering performance of A/CO_2_ and A/light curves.
**Fig. S2** Clustering of A/CO_2_ curves.
**Fig. S3** Clustering of A/light curves.
**Fig. S4** Detailed architecture of the surrogate neural network model.
**Fig. S5** Detailed architecture of the C4TUNE model.
**Fig. S6** Example of parameter sets resulting in identical $A_{\mathrm{net}}$ response curves.
**Fig. S7** Default performance scores of different classifier models for the prediction of infeasible parameter sets.
**Fig. S8** Most important features for the classification of parameter vectors based on feasibility.
**Fig. S9** Partial dependences of the most important features for the classification of parameter vectors based on feasibility.
**Fig. S10** Important features for the surrogate model predictions.
**Fig. S11** Comparison of *V*_max_ values determined by classical curve fitting and prediction by C4TUNE.
**Fig. S12** Example curve simulations based on predicted parameters using C4TUNE.
**Fig. S13** Example curve simulations based on predicted parameters for sampled $A_{\mathrm{net}}$ response curves within experimental error using C4TUNE.
**Fig. S14** Correlations between $A_{\mathrm{net}}$ and predicted parameter values for *Zea mays*.
**Fig. S15** Correlations between $A_{\mathrm{net}}$ and predicted parameter values for *Sorghum bicolor*.
**Fig. S16** Simulation results after optimization of selected model parameters.
**Fig. S17** Simulation results after optimization of selected model parameters.
**Fig. S18** Simulation results after optimization of selected model parameters.
**Fig. S19** Simulation results after optimization of selected model parameters across all genotypes.
**Fig. S20** Simulation results with increased *V*_max_ values for the RuBisCO carboxylation and oxygenation reactions.
**Fig. S21** Comparison of prediction performances between C4TUNE and classical machine learning approaches.
**Notes S1** Estimation of initial values for parameter sampling to generate the artificial dataset (Xu *et al*., 2025).
**Notes S2** Identification of parameters that determine the feasibility of model simulations.
**Notes S3** Identification of important parameters that determine the MSE of surrogate model predictions.
**Notes S4** Processing of gas exchange data from Almeida *et al*. (2025) for parameter prediction with C4TUNE.
**Notes S5** Compensation effects between parameter pairs.
**Notes S6** Identification of co‐limiting parameters on *A*_net_.
**Table S1** Compensating parameter pairs ranked by pairwise correlation and correlation to *A*_net_ across A/CO_2_ and A/light curves in the synthetic dataset.
**Table S2** Potentially co‐limiting parameter pairs ranked by log‐likelihood ratios.
Please note: Wiley is not responsible for the content or functionality of any Supporting Information supplied by the authors. Any queries (other than missing material) should be directed to the *New Phytologist* Central Office.

## Data Availability

The gas exchange measurements for maize genotypes are available at doi: 10.5281/zenodo.15966533. Part of these data has been used in another study linking photosynthesis‐related traits and hyperspectral reflectance data (Xu *et al*., 2026). The generated artificial data set for neural network training is available at doi: 10.5281/zenodo.15926601. Custom code for the generation of the artificial dataset as well as code for neural model definition and training is available at https://github.com/pwendering/C4TUNE. This repository also contains the predicted parameters for the maize genotypes.
